# Nutritional Quality, Antioxidant, Microstructural and Sensory Properties of Spontaneously Fermented Gluten-Free Finger Millet Biscuits

**DOI:** 10.3390/foods11091265

**Published:** 2022-04-27

**Authors:** Masala Mudau, Mpho Edward Mashau, Shonisani Eugenia Ramashia

**Affiliations:** Department of Food Science and Technology, Faculty of Science, Engineering and Agriculture, University of Venda, Thohoyandou 0950, Limpopo Province, South Africa; 11632442@mvula.univen.ac.za (M.M.); mpho.mashau@univen.ac.za (M.E.M.)

**Keywords:** fermentation, millet, proximate composition, polyphenols, organoleptic properties

## Abstract

Finger millet (FM) is a nutritious and gluten-free cereal grain which is rich in dietary fibre, minerals and antioxidant properties, thereby making it an ideal raw material for preparing gluten-free foods for people suffering from celiac disease. Spontaneous fermentation of FM grains has shown improved nutritional and functional properties of its flour and can be used as a functional ingredient for gluten free biscuits. The aim of this study was to determine the effect of spontaneous fermentation (SF) on the nutritional quality, antioxidant, microstructural, and sensory characteristics of gluten-free FM biscuits obtained from light and dark brown FM flours. Results showed that SF decreased ash, crude fibre, and crude fat contents as well as total phenolic and flavonoids contents. Protein content, carbohydrates content, energy values, antioxidant activity (DPPH and FRAP), and mineral content of FM biscuits increased due to SF. The colour properties such as lightness (L*), showed a significant increase as SF period increased in light brown FM biscuits, however dark brown FM biscuits showed no significant difference. The hue angle and colour differences (ΔE) of FM biscuits increased with the increasing period of SF, ranging from 43.20 to 53.76° and from 0.67 to 7.96, respectively. Spontaneous fermentation also decreased physical properties of biscuits such as diameter (4.76 to 4.54 cm), weight (12.77 to 11.99 g), and spread ratio (7.25 to 6.05), while an increase in thickness and hardness was noted. Spontaneous fermentation also induced changes on the microstructure of FM biscuits. Among the fermented biscuits, panelists preferred 24 h gluten-free fermented FM biscuits since they had better sensory properties. Overall, SF enhanced the nutritional value and health promoting compounds of gluten-free FM biscuits.

## 1. Introduction

Millets are cereals that belong to the Poaceae family of grasses. The name millet is derived from “mille”, a French word meaning a thousand, which signifies that thousands of grains can be found in a handful of millet [1]. Millets come in different varieties, including finger millet, pearl millet, little millet, barnyard millet, and kodo millet, also known as *Eleusine coracana*, *Pennisetum glaucum*, *Panicum sumatrense*, *Echinochloa Frumentacea* Link, and *Setara italic*, respectively [2,3]. Finger millet (FM), after sorghum, pearl millet, and foxtail millet, is the fourth most important millet in the world [4]. It is commonly grown in Africa and South Asia, and accounts for around 10% of the world’s 30 million tons of millet production, and this shows that the production of FM is sufficient for market application [5]. It is also a nutrient-dense grain, which is underutilised, neglected, and is commonly known as African millet, *ragi* in India and *mufhoho* in Venda, South Africa. Protein, dietary fibre, minerals, polyphenols, and antioxidants are all abundant in FM grains [6]. Finger millet is a rich source of calcium, potassium and manganese compared to other millets [7]. As compared to all cereals, FM contains the highest content of calcium [8]. The grains contain no gluten and have a very low glycaemic index, which is good for celiac patients as well as those with heart disease or hypertension [9,10].

Biscuits are an ideal flour-based food that can be consumed as snack food by young and elderly people [11]. They have been utilised as a vehicle to deliver important nutrients (proteins and vitamins), polyphenols, and antioxidants [12,13]. They have the advantages of being easily digestible and consumable. Among foods that are ready to eat, biscuits are significantly important because they are broadly accepted, cheap, and their shelf life is quite long [14]. Unlike bread, biscuits are small and easy to bake without gluten and still have good sensory attributes that are appealing to consumers [11]. They are produced by mixing several ingredients, such as flour, sugar, water, and fat to form a dough [15].

There is a growing need for gluten-free (GF) products in order to tackle the issue of celiac disease. Celiac disease is a systemic, immune-mediated enteropathy which is triggered by gluten protein in genetically susceptible individuals [16]. About 40–60 million people globally suffer from celiac disease, and it is only treated by consuming GF food products [17]. However, nutrient deficiencies in GF food products are a concern because most GF products are commonly made from refined flours that lack protein, dietary fibres and micronutrients such as minerals and vitamins [18]. Finger millet is high in micronutrients; thus, it can be used as a potential material to make micronutrient-rich GF products to address nutritional challenges in GF diets [19,20]. With poor accessibility of minerals of plant-based diets, processing technologies such as spontaneous fermentation can be utilised to improve the bioavailability of micronutrients such as calcium, iron, and zinc, as well as polyphenols and antioxidants activity [21,22]. Research shows that spontaneous fermentation (SF) reduces antinutritional factors and enhances the bio-accessibility of nutrients [23,24]. Moreover, SF has been reported to improve protein content and dietary fibre in pearl millet bread and foxtail flour, respectively [25,26].

Lactic acid bacteria are some of the microorganisms that have been reported to bring desirable changes in FM flours [27]. Furthermore, lactobacillus and pediococcus sp are used as fermenting microorganisms in the production of Indian fermented FM food product (*Koozhu*), and this shows the safe use of fermented FM flour as an ideal substrate for the development of functional foods [28]. Modification in the microstructure, proximate composition, mineral composition, polyphenols, and sensory acceptance of pearl millet biscuits due to SF have also been reported [14,29,30]. However, little is known about how different periods of SF affects nutritional composition, polyphenols, antioxidant activity, physical, microstructural properties, and sensory acceptability of gluten-free FM biscuits. Hence, the objective of this study was to examine the nutritional quality, antioxidant, microstructural and sensory properties of spontaneously fermented gluten-free FM biscuits.

## 2. Materials and Methods

### 2.1. Materials

Two cultivars (light and brown) of FM grains as well as additional baking ingredients (sunflower oil, baking powder, vanilla essence, and sugar) for biscuits production were purchased from local street vendors and the Shoprite supermarket in Thohoyandou, Limpopo province, South Africa. All analytical grade reagents and standards (boric acid, quercetin standard, Folin-Ciocalteu reagent, 2,2-diphenyl-lpicrylhydrazyl (DPPH), gallic acid standard, trichloroacetic acid) used were procured from Merk chemicals (PTY) Ltd., Germiston, South Africa.

### 2.2. Finger Millet Flour Production

The grain chaffs and other unwanted materials were removed through winnowing, sorting, and screening. The grains were then washed with clean water and dried (40 °C) for 24 h using an air oven drier. After drying, some of grains were ground pulverised (ZM 200 Miller, Retsch, Düsseldorf, Germany) to obtain native flour, while the remaining grains were kept for the SF process. The native flour was sifted with a 500 µm sieve, placed in a polyethylene bag and refrigerated at 4 °C [31].

### 2.3. Finger Millet Fermentation

Fine fermented FM flours were obtained using the modified method of Adebiyi et al. [14]. Approximately 400 g of FM grains were steeped in 1600 mL of distilled water in a closed container and left to spontaneously ferment at a temperature of 28 °C for 24, 48 and 72 h. After each fermentation period, the water was discarded, and the wet fermented grains were oven dried (40 °C) for 24 h. After that, the fermented grains were ground into (ZM 200 Miller, Retsch, Düsseldorf, Germany) flours, which was sieved with 500 µm sieve to obtain the fermented FM flours.

### 2.4. Biscuit Production

The FM biscuits were produced following a slightly modified method used by Serrem et al. [32]. The recipe for biscuits consisted of 112.5 g FM flour, sunflower oil (33 g), sugar (28 g), baking powder (0.75 g), vanilla essence (6.75 g) and water (60 g), which were blended well in a bowl to form a dough, which was then carefully rolled by hand to the desired thickness (0.7 cm) and cut with a circular-shaped biscuit cutter (5.3 cm diameter). The moulded dough was then placed in an oven (Defy, Model DSS700, Midrand, South Africa) and baked (180 °C) for 18 min. Baked products were cooled down for 20 min at room temperature before being packed in plastic and kept in a refrigerator until further analysis. The entire production of biscuits and analyses were replicated three times to validate the results, and nine biscuits per formulation were analysed. Biscuits made from native flours were used as control.

### 2.5. Proximate Composition of Fermented Finger Millet Flours and Biscuits

The AOAC [33] methods: 934.01, 978.10, 923.03, 920.39, and 990.03 were used to determine moisture, crude protein, ash, crude fat, and the crude fibre of FM flours and biscuits. The carbohydrate percentage of the flour and biscuit samples was calculated using the formula:

Carbohydrate (g) = 100 − [protein (g) + moisture content (g) + lipid (g) + ash (g)+ crude fibre (g)]

The energy content of FM flours and biscuits was obtained by using the formula:

Energy content (kcal) = g/100 g carbohydrate × 4 + g/100 g fat × 9 + g/100 g protein × 4 + g/100 g fibre × 2

### 2.6. Mineral Analysis of Fermented Finger Millet Biscuits

The macro and micro-minerals of FM biscuits were assessed following a method used by Ramashia et al. [34]. Milled biscuits samples (2 g) were burned in the muffle furnace overnight at 550 °C to obtain ashes. The obtained ashes were treated with 50% HCL (10 mL) and 5 mL of 33% HNO_3_ and the combination was allowed to stand for 1 h in the water bath. Afterward, HCL (10 mL) was added to the mixture and left in the water bath for 15 min, before it was transferred into a 100 mL volumetric flask. Thereafter, distilled water was added into the 100 mL volumetric until the 100 mL mark was reached. The mixture was placed in the water bath for 1 h. Approximately 10 mL of HCL was added and left in a water bath for 15 min. The mixture was then added into a 100 mL volumetric flask and filled to the 100 mL mark with distilled water before it was well mixed. Inductively coupled argon plasm atomic emission spectroscopy was used to analyse the minerals and the obtained results were expressed as mg/100 g.

### 2.7. Polyphenols and Antioxidant Activity of Fermented Finger Millet Biscuits

Extraction of polyphenols, DPPH and iron reducing power was determined by mixing 50 g each of flour with methanol (500 mL) for 24 h. Thereafter, the combination was subjected to centrifugation (Rotina 380 R, Labotech Ecotherm, Midrand, South Africa) for 10 min at 3000 rpm, filtered using Whatman paper into beakers and transferred into different centrifuge tubes which were kept in the freezer for further analysis [35].

#### 2.7.1. Total Phenolic Content (TPC)

The TPC of FM biscuits extract was examined following a method employed by Demov et al. [36] with slight modifications. An extract (0.2 mL) was combined with a mixture of five times diluted Folin-Ciocalteu (2.5 mL) and 5 mL distilled water in test tubes. After 5 min, 15% sodium carbonate (7.5 mL) was added to the tubes, vortexed (Model 36110740, Separation Scientific, Roodepoort, South Africa), and the mixture was stored in the dark for 30 min. A spectrophotometer (UV-1600, Shimadzu, Tokyo, Japan) was used to the record the values of absorbance at 760 nm. The standard curve was prepared with gallic acid and obtained results were expressed in milligrams of gallic acid per gram of biscuit sample.

#### 2.7.2. Total Flavonoid Content (TFC)

The TFC of FM biscuits extract was examined following a slightly modified method described by Mahloko et al. [37]. Biscuit sample extract was combined with 5% NaNO_2_ (0.3 mL) in a tube and the mixture reacted for 5 min prior to the addition of 10% AlCl_3_ (0.6 mL). After 6 min, distilled water and 1 M NaOH (2 mL) were added and vortexed. A spectrophotometer (UV-1600, Shimadzu, Tokyo, Japan) was employed to record the values of absorbance at 510 nm. The quercetin standard ($R^{2}$ = 0.9992) was used for standard curve and obtained results were measured in milligrams of Quercetin per gram of biscuit sample.

#### 2.7.3. DPPH Radical Scavenging

The DPPH assay of FM biscuit was analysed as described by De Ancos et al. [38], wherein 3.9 mL 0.1 mM DPPH was added to the mixture of biscuit extract (10 µL) and distilled water (90 µL). The combination was thoroughly mixed before being left in the dark to react for 30 min, after which the absorbance of the mixture was measured at 517 nm using a spectrophotometer (UV-1600, Shimadzu, Tokyo, Japan).

#### 2.7.4. Ferric Reducing Antioxidant Power (FRAP)

A method used by Lou et al. [39], was employed to measure the FRAP assay of FM biscuit samples. Biscuits’ sample extract (100 µL) and methanol were mixed in a test tube to make a volume of 1 mL. The content was blended with 0.2 M phosphate buffer and 1% K_3_[Fe (C N)_6_] (2.5 mL) and mixed well and then centrifuged (Rotina 380 R, Labotech Ecotherm, Midrand, South Africa) at 5000 rpm for 20 min. The obtained supernatant was combined with a mixture of 0.1 mM FeCL_3_ (1 mL) and distilled water (1 mL). The absorbance of the combination was measured with a spectrophotometer (UV-1600, Shimadzu, Tokyo, Japan) at 700 nm. A greater absorbance combination suggested a higher reducing power.

### 2.8. Physical Properties of Fermented Finger Millet Biscuits

#### 2.8.1. Colour of Biscuits

The top surface colour attributes (L*, a* and b*) of FM biscuits was analysed using a Hunter Lab colorimeter (MiniScan XE Plus, Model CM-3500d, Hunter Associate laboratory, Reston, VA, USA) with a D65 light source. Before the analysis, the colorimeter was calibrated. The values for L*, a* and b* expressing the colour readings were used to calculate the chroma (C), hue angle (H°), and colour change (ΔE) using the following formulas:

$$\mathrm{Chroma}=\sqrt{{\left(a^{*}\right)}^{2}+{\left(b^{*}\right)}^{2}}\;\mathrm{Hue}\;\left(H^{{}^{\circ}}\right)=\tanh^{-1}\{\frac{b^{*}}{a^{*}}\}\;\mathrm{The}\;\mathrm{total}\;\mathrm{colour}\;\mathrm{difference}\;\left(∆E\right)=\sqrt{{\left(L-\mathrm{Lc}\right)}^{2}+{\left(a-\mathrm{ac}\right)}^{2}+{\left(b-\mathrm{bc}\right)}^{2}}$$

where L = lightness, a = redness, b = yellowness, Lc = lightness of control sample, ac = redness of control sample, bc = yellowness of control sample.

#### 2.8.2. Thickness, Diameter, Weight and Spread Ratio

A caliper was utilised to measure the thickness and diameter of each of the biscuit samples and biscuits were randomly selected. An average of nine values was taken for each batch of biscuit samples. The average value for thickness and diameter was reported in centimeters. A weighing balance was used to determine the weight of each of nine biscuit samples per formulation. The average value for weight was reported in grams. The biscuits’ spread ratio was obtained by dividing the biscuit diameter by the biscuit thickness.

#### 2.8.3. Texture

The TA-XTplus texture analyser (stable Micro System, Surrey, UK) was used to assess the hardness of FM biscuits. A 5 kg load cell, a 3-point bend ring and a heavy-duty platform were used. The automatic settings for test speed and trigger force were 3.0 mm/s and 50 g, respectively. The peak force was recorded as the hardness value of the biscuits [40].

### 2.9. Microstructural Analysis of Fermented Finger Millet Biscuits Using Scanning Electron Microscopy (SEM)

Scanning electron microscopy (Model, JSM 6610-LV, Chicago, IL, USA) at magnification of ×1000 and scale bar of 20 µm was used to examine the morphological characteristics of FM biscuits. The samples were placed on an aluminium stab with the aid of cellophane tape and coated using gold in an auto fine coater (JEO-JFC-1600) [41].

### 2.10. Sensory Evaluation of Fermented Finger Millet Biscuits

The University of Venda internal Ethics Committee granted ethical clearance (SEA/21/FST/08/1214) to the investigator before sensory evaluation was conducted. Consumer evaluation of FM biscuits amid COVID-19 was conducted in the lecture hall using university consumers (staff and students) (*n* = 60). Panelists were asked to follow all COVID-19 safety protocols before the briefing about the product and evaluation of biscuit samples for appearance, colour, aroma, taste, texture, and overall likeness using a 9-point hedonic structural scale (where 1 = dislike very much, 5 = neither like nor dislike and 9 = like extremely). The biscuit samples (8 pieces each) were presented in a white disposable plate covered with aluminium foil. Panelists were requested to taste the samples according to the plate presentation order and were required to rinse their mouths with tap water for 1 min before and after each test.

### 2.11. Statistical Analysis

The assessment of data was done by analysis of variance (ANOVA) in SPSS 26 for windows (SPSS Inc., Chicago, IL, USA). The Duncan’s Multiple Range Test (*p* ≤ 0.05) was done to compare mean values.

## 3. Results and Discussion

### 3.1. Proximate Composition of Spontaneously Fermented Finger Millet Biscuits

Table 1 shows the effect of SF on the proximate composition of FM flours and biscuits. The moisture content of light and dark brown FM flours decreased significantly (*p* < 0.05) with increasing period of fermentation. When fermentation time increases, compact polymers become simpler, making it difficult to bind water, resulting in the easily evaporation of water during drying [42]. A similar decrease of moisture content was observed by Adebiyi et al. [14] for spontaneously fermented pearl millet flours. There was no significant difference in the moisture content of biscuits (*p* < 0.05). The ash content of FM and biscuits decreased as SF time increased. Higher ash content values were obtained in native FM flours and biscuits while low ash values were obtained in 72 h fermented FM flours and biscuits. The decrease in ash content of both flours and biscuits was probably due to either the leaching of mineral elements that are soluble into the fermenting medium (acid liquid) or the fermenting microorganisms’ general activities which include the use of ash related components for metabolism [31]. Many studies have also reported a decrease of ash contents due to SF [14,22,43].

The fibre content of light and dark brown FM flours and biscuits showed a decrease as SF time increased. Higher fibre content values were obtained in native FM flours and biscuits while low fibre content values were obtained in 72 h fermented FM biscuits. During SF, there are extracellular enzymes produced by microbes that are capable of hydrolysing and metabolising insoluble polysaccharide. The β-D-glucosidase hydrolyses the terminal part of polysaccharide chains which results in the decrease of crude fibre of fermented products, hence the decrease of crude fibre in the fermented biscuits [44]. Again, according to Tefere et al. [44] fermentation tends to reduce soluble fibre more than insoluble fibre, which further contributes to the reduction in crude fibre. This is congruent with a report by Azeez et al. [45], who observed a decrease of crude fibre after fermentation.

The carbohydrate content of light and dark brown FM flours and biscuits increased with the increasing period of SF. de Olivera Silva et al. [46] observed a similar increase of carbohydrate due to SF.

The protein content of both flours and FM biscuits increased as SF time increased. The highest protein contents were obtained in 72 h fermented FM samples. A similar trend of increase of protein after fermentation has been observed by Longeria et al. [47]. The enhancement of protein content could be ascribed to the accumulation of proteins in the form of extracellular enzymes produced by lactic acid bacteria during fermentation. This is corroborated by Siezen & van Hylckama Vlieg [48], who reported the production of proteinaceous enzymes by lactic acid bacteria during fermentation, hence the increment of protein content in the fermented flours and biscuits. Other researchers have attributed the protein increment to synthesis of proteolytic enzymes during SF, which hydrolyses proteins into amino acids and peptides [49,50].

The fat content of both flours and biscuits decreased as fermentation time increased. The highest fat content was found in native FM flours and biscuits, while lower fat content was found in 72 h fermented FM flours and biscuits. The fat values obtained in native biscuits were similar to those reported by Mehra and Singh [51] for biscuits prepared from pearl millet flour. The reduction of fat in the fermented samples could be attributed to lipolytic enzyme activity increase, which degrades fat components into glycerols and fatty acids through hydrolysis [22]. The increment of fat content of FM biscuits as compared to FM flours was due to the addition of fat during dough preparation. Spontaneous fermentation also increased the energy content values of light brown and dark brown FM flours and biscuits. The increment in the energy content of FM flours observed was probably due to an increase in protein and carbohydrate contents, as these parameters also contribute to energy contents [22]. It could also be due to the decrease in fibre content caused by fermentation. According to Hervik [52], fermented fibre contributes to energy value by producing short-chain fatty acids which act as a source of energy. Nevertheless, a significant decrease in the energy content of 72 h fermented FM biscuits could be attributed to the decrease in fat content.

### 3.2. Mineral Content of Fermented Finger Millet Biscuits

The mineral composition of fermented light and dark brown FM biscuits is presented in Table 2. In light and dark brown FM biscuits, the mineral content increased with the increasing period of SF. Biscuits obtained from 72 h fermented flours contained significantly (*p* < 0.05) higher values of macro and micro-minerals compared to the mineral contents obtained in biscuits made from native flours. In native flour, minerals can form insoluble complexes with antinutritional factors including phytates and tannins, which can be broken during SF, resulting in the increased bioavailability of minerals [53].

This may explain the increase of mineral content in fermented biscuits. The mechanisms behind the liberation of minerals from phytate could be via the dephosphorylation of phytate, whereby phosphate groups are removed from the inositol ring resulting in the decrease of the phytate’s mineral binding strength, which enhances the bioavailability of minerals [54]. The higher improvements of minerals such as copper, iron, manganese, and zinc observed in this study could be more closely linked to SF. Chidera [55] ascribed the increase in the macro-minerals such as phosphorus to its liberation from organic complex caused by microflora enzymes. These observed increases of minerals with increasing period of SF agree with finding by Banwo et al. [56], whereby fermenting microorganisms enhanced the quality of FM and sorghum gruels when the minerals bioavailability increased. Mineral deficiency is a shortage of dietary minerals, which are essential for good the health of humans, and it is caused by a poor diet, impaired intake of minerals after consumption or poor utilisation of minerals [57]. Calcium deficiency, iron deficiency and zinc deficiency are among the examples of mineral deficiencies that are negatively affecting the health of people. A shortage of calcium in the body has been reported to cause osteoporosis, osteomalacia, and rickets, especially in Africa and Asia, while an insufficient supply of iron lead to anemia [58]. The deficiency of zinc causes stunting, diarrhea, and pneumonia in infants and children. Therefore, the improved mineral content of fermented FM biscuits can help eradicate mineral deficiency, especially in children, if they are consumed regularly.

### 3.3. Polyphenols and Antioxidant Activity of Spontaneously Fermented Finger Millet Biscuits

Table 3 shows the influence of SF on polyphenols and the antioxidant activity of fermented FM biscuits. Unlike in previous studies [59,60], TPC and TFC in this study decreased as SF time increased. The highest TPC and TFC were found in native FM biscuits, while the lowest TPC was found in 72 h fermented FM biscuits. The decrease in TPC and TFC could be attributed to the fermenting microflora’s polyphenol oxidase activity [61]. It could also be attributed to the abstraction of the hydride ion and phenolic structure rearrangement caused by the acidic environment during fermentation [62]. A similar trend of decrease in TPC after SF was observed by Adebiyi et al. [14] for GF pearl millet biscuits.

The scavenging activities (DPPH) of light and dark brown FM biscuits increased significantly with the increasing period of SF. The highest DPPH was obtained in 72 h fermented FM biscuits, while the lowest DPPH was obtained in native FM biscuits. The higher the DPPH value of biscuits, the stronger the scavenging activity of the sample. The increase in DPPH observed after SF of FM biscuits could be attributed to the liberation of more soluble bioactive compounds, including oligosaccharides and peptides produced during fermentation [63]. Similar findings whereby the release of more soluble or easily extractable phenolics caused by SF which led to an increase in the DPPH in the sough dough were reported by Liukkonen et al. [64]. Srivastava et al. [65] investigated how SF affects the antioxidant activity of pearl millet flour and found that SF decreased the TPC while increasing DPPH.

Spontaneous fermentation also influenced the iron reducing activity, as a significant increase was observed up to 48 h SF time in both light and dark brown FM biscuits. However, a decrease in the iron reducing activity of 72 h fermented FM biscuits as compared to other fermented FM biscuits was observed. Because TPC, TFC and antioxidant activity have a negative correlation, the increase of antioxidant activity could be due to protein structural changes induced by SF. Nissen et al. [66] found that protein is involved in the enhancement of antioxidant activities by producing peptides after baking, which has stronger activity. Free sugars and protein components such as amino acids produced during fermentation boost Maillard reactions during baking, releasing additional high antioxidant-containing compounds such as melanoidin and reductones [67]. Therefore, the possibility of an increased iron reducing activity due to conformational protein changes during SF and baking cannot be ruled out.

Heat disrupts the cell wall, allowing more antioxidant compounds to be released, resulting in the increase of antioxidant activity [68]. Hussain et al. [69] also found the increment of antioxidant activity of wheat-millet biscuits that was caused by some compounds produced by the Maillard reaction during baking. Higher antioxidant activity obtained in fermented FM biscuits enhanced their health benefits. Antioxidants are said to act as lipid stabilizers and suppressors of excessive oxidation, which is linked to cancer and aging [70].

### 3.4. Colour Attributes of Spontaneously Fermented Finger Millet Biscuits

**Figure 1:**
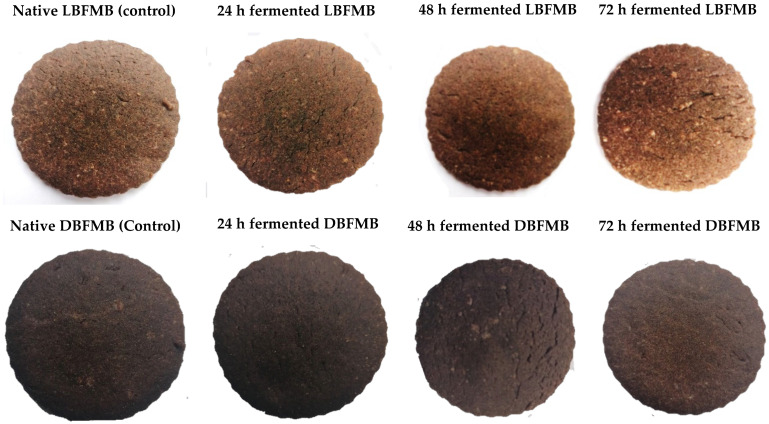
Images of finger millet biscuits. LBFMF = light brown finger millet biscuit, DBFMB = dark brown finger millet biscuit.

Biscuit colour, as shown in Figure 1, is largely determined by the amount of browning that occurs during baking which is influenced by the amount of dissolved reducing sugar as well as the amount of water used [71]. Table 4 shows the colour of light and dark brown fermented FM biscuits. The L* values of light brown FM biscuits increased significantly as SF time increased. A similar increasing trend in L* values was also found by Okin et al. [72] for biscuits made from fermented sorghum flour. As for dark brown FM biscuits, there was no significant difference in terms of the L* values.

In terms of the a* values, which represent the redness of the biscuits, there was no significant difference observed. The yellowness (b*) of light brown FM biscuits increased as the SF time increased, while for dark brown FM biscuits, there was no significant difference observed.

The chroma value for light brown FM biscuits increased with the increasing period of SF, while for dark brown FM biscuits, there was no significant difference observed. Hue angle is a qualitative colour property on the basis of colours such as greenish, reddish, and yellowish [73]. Red hue is represented by a hue angle of 0° or 360°, while yellow, green, and blues are represented by angles of 90°, 180° and 270°, respectively. All the biscuits had Hue values of less than 90°, indicating that they were less yellow in the CIE-LAB colour space and had a brown spectrum in the visible region of the opponent colour chart.

Regarding the total colour difference (ΔE), a lower ΔE value of 0.67 obtained in 24 h dark brown fermented biscuits indicate that SF had no effect on the colour of the biscuits, whereas higher ΔE values (1.26 & 7.96) obtained in 72 h fermented FM biscuits suggest that SF had a negative impact on the colour of the biscuits.

### 3.5. Physical Properties of Spontaneously Fermented Finger Millet Biscuits

Table 5 shows the physical characteristics of FM biscuits. The results show that SF did not have any significant effect on the diameter and thickness of the biscuits. The weight of light and dark brown biscuits decreased as SF time increased. A higher weight was obtained in biscuits made from native FM flours. High fibre content restricts more moisture loss during the process of baking, which results in the higher weight of the biscuits [74]. This is in line with a report by Omoba et al. [69] who indicated that higher fibre content is related to higher weight of biscuits. The decrease of weight in fermented biscuits could be due to the decrease in the fibre contents of flours (Table 1) during SF.

Spread ratio or diameter is determined by the flours’ quality utilised in the preparation of biscuits as well as the biscuits’ ability to expand [75]. The spread ratio is mostly governed by the protein content of the flours [76]. The spread ratio results for light brown FM biscuits were lower in 48 and 72 h fermented biscuits. In dark brown FM biscuits, the spread ratio decreased with the increasing period of fermentation. The decrease of the spread ratio observed in fermented biscuits could be due to the protein content increase caused by SF. Oyeyinka et al. [77] indicated that high protein content flours produce biscuits with a low spread ratio. Simply put, the higher the protein in the dough, the more water that is retained, and the dough’s viscosity increases, which results in a low spread ratio of the biscuits [76]. This explanation is also corroborated by Alioglu & Ozulku [78], who linked high dough protease or proteinase activity with a lower spread ratio. Similarly, Oyeyinka et al. [77] discovered that cookies made from fermented powdered cassava with a high protein content had a lower spread ratio.

Texture, along with visual appearance, taste, and aroma, are important attributes of the sensory quality of food [79], and their analysis and evaluation are critical during new food product development. The hardness of light and dark brown FM biscuits increased significantly with an increasing period of SF. Basically, SF increases the hardness of biscuits and Kulthe et al. [80] ascribed it to enhanced protein-starch interaction via hydrogen bonding. It could also be due to the increase in the thickness of the biscuits. Alioglu & Ozulku [78] reported that a decrease in the amount of water in the fermented dough because of the acids produced during starch hydrolysis can lead to the development of new linkages between carbohydrates, free amino acids and denatured protein, which increases the hardness of the biscuits. Similar hardness values were also obtained by Sulieman et al. [74] for GF-baked products made from fermented *Agaricus bisporus* polysaccharide flour.

### 3.6. Microstructural Properties of Spontaneously Fermented Finger Millet Biscuits

Figure S1 (Supplementary Materials) shows the morphological structures of spontaneously fermented light and dark brown FM biscuits. The images of native light and dark brown FM biscuits showed a matrix of protein and lipid in which some starch granules (SG) of different sizes ranging from small to medium were embedded. Similarly, Filipčev et al. [81] described the microstructure of biscuits as a matrix of lipid and protein in which SG are embedded. In the fermented FM biscuits (24 & 48 h), SG were still clearly not visible. However, the SG increased as the fermentation period further increased.

The formation of holes on the SG surface was observed in the 72 h fermented biscuits as they were also observed in other fermented biscuits. This could be attributed to the decomposition of fibre (Table 1) by fermentation, which caused the cell membrane to rupture easily when the gas expansion increased during baking, letting gas escape through the pores. The SG after 72 h of fermentation formed a honeycomb like structure as was also observed by Adebiyi et al. [29] for spontaneously fermented GF biscuits made from pearl millet flour.

### 3.7. Sensory Evaluation of Spontaneously Fermented Finger Millet Biscuits

Table 6 shows the organoleptic evaluation of light and dark brown FM biscuits. According to the appearance mean scores, SF did not have any significant effect on the panelists perception of biscuit appearance. The colour mean scores for 48 and 72 h fermented biscuits were significantly (*p* < 0.05) lower than those for 24 h and native biscuits. Panelists disliked the colour of 48 and 72 h fermented dark brown FM biscuits. This could be due to dark colour that was observed, which may have given the panelists the impression that biscuits were over-baked, influencing their preference. Aroma is the most important component that determines whether a product is accepted or not [82]. The panelists found the aroma of native and 24 h fermented FM biscuits more appreciable than that of 48 h and 72 h fermented biscuits. In terms of taste, light brown FM biscuits had a higher score than dark brown FM biscuits. However, native biscuits had higher scores than fermented biscuits. The low mean scores for taste of 72 h fermented biscuits, especially dark brown FM biscuits, could be attributable to high amounts of antioxidants and nutraceuticals and lactic acid produced during SF which might have also caused the biscuits to have an unpleasant aroma [83,84]. The panelists described the aroma and taste of the fermented biscuits as bad and bitter. The findings in this study regarding aroma and taste are comparable to those of de Oliveira Silva et al. [46] and Oyeyinka et al. [77] who found low mean scores for the aroma and taste of biscuits made from fermented soybean flours and cassava flours. Regarding texture and overall acceptability, light brown FM biscuits were better than dark brown FM biscuits. Biscuits made from 72 h fermented light and dark brown scored the lowest in terms of texture and overall acceptability. The low mean scores for overall acceptability of 72 h fermented biscuits could be ascribed to the bad aroma and bitter taste associated with them.

## 4. Conclusions

In this study, SF was used as a conventional and natural means to improve the nutritional composition, health promoting compounds and microstructural properties of GF FM biscuits. Spontaneous fermentation increased the protein content, carbohydrate content, energy values, mineral content, and antioxidant capacity of gluten free FM biscuits. The improved mineral content of GF FM biscuits means that daily consumption of FM biscuits can prevent mineral deficiencies in children in third world countries such as South Africa. In terms of sensory acceptance of GF FM biscuits, panelists preferred 24 h fermented biscuits compared to the rest. Spontaneous fermentation is a successful strategy to provide consumers with GF FM biscuits that are nutritious and rich in antioxidants. Further studies should be conducted on the impact of SF on amino acids, protein quality, antinutritional factors and mineral extractability of GF FM biscuits or on other products that can be produced from FM flours.

## Figures and Tables

**Table 1:** Effect of fermentation time on the proximate composition of finger millet flours and biscuits (% dry basis).

Fermentation Period (h)	Moisture	Ash	Crude Fibre	Protein	Crude Fat	Carbohydrates	Energy (kcal/100 g)
Light brown FM flours							
0	11.56 ± 0.10 ^a^	2.49 ± 0.06 ^a^	2.91 ± 0.05 ^a^	7.87 ± 0.42 ^a^	1.82 ± 0.03 ^a^	73.34 ± 0.12 ^a^	347.04 ± 0.80 ^a^
24	10.69 ± 0.17 ^b^	1.77 ± 0.04 ^b^	2.81 ± 0.02 ^b^	8.29 ± 0.08 ^b^	1.72 ± 0.02 ^b^	74.73 ± 0.13 ^b^	353.18 ± 0.77 ^b^
48	10.43 ± 0.11 ^c^	1.53 ± 0.03 ^c^	2.71 ± 0.02 ^c^	8.67 ± 0.04 ^c^	1.62 ± 0.04 ^c^	75.04 ± 0.08 ^c^	354.80 ± 0.61 ^c^
72	9.65 ± 0.15 ^d^	1.10 ± 0.01 ^d^	2.53 ± 0.03 ^d^	9.09 ± 0.05 ^d^	1.53 ± 0.02 ^d^	76.10 ± 0.23 ^d^	355.72 ± 0.68 ^d^
Dark brown FM flours							
0	11.78 ± 0.11 ^a^	2.12 ± 0.01 ^a^	3.11 ± 0.12 ^a^	8.00 ± 0.04 ^a^	1.75 ± 0.06 ^a^	73.23 ± 0.09 ^a^	346.76 ± 0.71 ^a^
24	11.42 ± 0.04 ^b^	1.76 ± 0.03 ^b^	2.89 ± 0.04 ^b^	8.41 ± 0.04 ^b^	1.67 ± 0.03 ^b^	73.84 ± 0.13 ^b^	349.71 ± 0.26 ^b^
48	10.95 ± 0.13 ^c^	1.46 ± 0.10 ^c^	2.74 ± 0.04 ^c^	8.82 ± 0.04 ^c^	1.52 ± 0.02 ^c^	74.51 ± 0.08 ^c^	351.83 ± 0.60 ^c^
72	10.48 ± 0.06 ^d^	1.38 ± 0.07 ^c^	2.63 ± 0.03 ^d^	9.27 ± 0.03 ^d^	1.45 ± 0.02 ^d^	74.79 ± 0.14 ^d^	354.54 ± 0.52 ^d^
Light brown FM biscuits							
0	6.91 ± 0.14 ^a^	1.66 ± 0.09 ^a^	3.11 ± 0.27 ^a^	9.16 ± 0.37 ^a^	22.11 ± 1.45 ^a^	57.05 ± 0.64 ^a^	470.04 ± 10.46 ^b^
24	6.83 ± 0.24 ^a^	1.39 ± 0.03 ^c^	3.03 ± 0.23 ^ab^	9.89 ± 0.57 ^ab^	21.48 ± 0.86 ^a^	57.38 ± 1.32 ^ab^	468.46 ± 11.34 ^b^
48	6.44 ± 0.67 ^a^	1.24 ± 0.03 ^cd^	2.89 ± 0.23 ^a^	10.71 ± 0.57 ^bc^	20.83 ± 1.11 ^ab^	57.89 ± 1.07 ^ab^	467.65 ± 10.94 ^b^
72	5.93 ± 0.14 ^a^	0.93 ± 0.06 ^d^	2.71 ± 0.15 ^a^	11.54 ± 0.71 ^c^	18.83 ± 0.76 ^b^	60.04 ± 0.97 ^b^	461.21 ± 10.36 ^a^
Dark brown FM biscuits							
0	7.60 ± 0.35 ^a^	1.52 ± 0.073 ^a^	3.45 ± 0.25 ^a^	9.88 ± 0.78 ^a^	20.75 ± 0.85 ^a^	56.79 ± 2.36 ^a^	460.33 ± 17.19 ^b^
24	6.44 ± 0.45 ^a^	1.32 ± 0.03 ^b^	3.20 ± 0.09 ^ab^	10.43 ± 0.47 ^ab^	19.58 ± 0.49 ^a^	58.03 ± 0.59 ^ab^	456.46 ± 7.92 ^b^
48	6.84 ± 0.89 ^a^	1.25 ± 0.04 ^bc^	2.96 ± 0.05 ^b^	11.48 ± 0.29 ^b^	18.11 ± 0.86 ^b^	59.36 ± 0.32 ^ab^	452.27 ± 4.57 ^ab^
72	6.67 ± 2.26 ^a^	1.17 ± 0.04 ^c^	2.80 ± 0.10 ^b^	11.66 ± 1.05 ^b^	16.69 ± 1.07 ^b^	61.01 ± 1.82 ^b^	446.49 ± 13.09 ^a^

Values are presented as means ± standard deviation. Different letters in the same column are significantly different (*p* < 0.05). FM = Finger millet.

**Table 2:** Influence of spontaneous fermentation on macro and micro-minerals content of finger millet biscuits. (mg/100 g dry basis).

LBFMB	Fermentation Period (h)			
Macro Elements	0	24	48	72
Ca	382.87 ± 1.42 ^a^	386.35 ± 1.05 ^b^	398.39 ± 0.41 ^c^	411.33 ± 1.99 ^d^
P	275.33 ± 1.23 ^a^	283.41 ± 0.96 ^b^	291.57 ± 0.97 ^c^	299.69 ± 1.88 ^d^
K	404.04 ± 1.69 ^a^	410.10 ± 1.27 ^b^	413.65 ± 1.06 ^c^	418.50 ± 0.82 ^d^
Mg	129.77 ± 1.76 ^a^	134.82 ± 1.19 ^b^	140.85 ± 0.81 ^c^	147.94 ± 1.06 ^d^
Na	7.37 ± 0.95 ^a^	9.61 ± 1.16 ^b^	12.25 ± 1.07 ^c^	16.84 ± 1.06 ^d^
Trace elements				
Cu	0.98 ± 0.15 ^a^	1.86 ± 0.24 ^b^	2.29 ± 0.14 ^c^	2.74 ± 0.18 ^d^
Zn	2.38 ± 0.76 ^a^	3.87 ± 0.87 ^b^	5.22 ± 0.80 ^b^	5.91 ± 0.91 ^c^
Fe	5.69 ± 0.92 ^a^	7.62 ± 0.62 ^b^	10.13 ± 0.67 ^c^	13.22 ± 0.56 ^d^
Mn	4.74 ± 0.59 ^a^	7.10 ± 1.16 ^b^	12.25 ± 1.07 ^c^	16.84 ± 1.06 ^d^
DBFMB				
Macro elements				
Ca	317.85 ± 1.33 ^a^	335.42 ± 1.92 ^b^	339.12 ± 1.49 ^c^	344.88 ± 1.13 ^d^
P	337.91 ± 1.84 ^a^	349.92 ± 0.99 ^b^	358.21 ± 1.33 ^c^	364.97 ± 1.10 ^d^
K	377.70 ± 1.12 ^a^	388.70 ± 0.95 ^b^	399.61 ± 0.59 ^c^	410.01 ± 0.41 ^d^
Mg	139.13 ± 1.28 ^a^	148.04 ± 0.64 ^b^	167.60 ± 0.42 ^c^	174.42 ± 1.09 ^d^
Na	4.86 ± 0.66 ^a^	9.46 ± 0.97 ^b^	14.64 ± 1.12 ^c^	20.29 ± 1.01 ^d^
Trace elements				
Cu	0.71 ± 0.12 ^a^	1.08 ± 0.24 ^b^	1.42 ± 0.12 ^b^	1.79 ± 0.78 ^c^
Zn	1.68 ± 0.21 ^a^	2.43 ± 0.30 ^b^	2.65 ± 0.12 ^c^	3.39 ± 0.61 ^d^
Fe	6.00 ± 0.95 ^a^	7.18 ± 0.64 ^a^	13.11 ± 0.79 ^b^	16.03 ± 1.13 ^c^
Mn	16.62 ± 1.15 ^a^	21.19 ± 0.32 ^b^	25.16 ± 0.70 ^c^	29.20 ± 1.02 ^d^

Different superscripts in the same rows are significantly different (*p* < 0.05). LBFMB = light brown finger millet biscuits; DBFMB = dark brown finger millet biscuits.

**Table 3:** Polyphenols and the antioxidant capacity of spontaneously fermented finger millet biscuits (dry basis).

Fermentation Period (h)	TPC (mg GAE/g)	TFC (mg QE/g)	DPPH (%)	FRAP (mg GAE/g)
Light brown FM biscuits				
0	58.78 ± 6.96 ^a^	3.11 ± 0.24 ^b^	52.78 ± 1.31 ^a^	0.7670 ± 0.09 ^a^
24	45.07 ± 4.35 ^b^	3.09 ± 0.31 ^b^	67.67 ± 5.30 ^b^	0.9077 ± 0.03 ^c^
48	37.22 ± 7.63 ^b^	3.02 ± 0.05 ^a^	76.42 ± 0.85 ^c^	0.9480 ± 0.01 ^d^
72	21.93 ± 3.56 ^c^	2.97 ± 0.02 ^a^	81.43 ± 1.23 ^d^	0.8270 ± 0.04 ^b^
Dark brown FM biscuits				
0	50.67 ± 0.97 ^b^	3.18 ± 0.02 ^c^	59.66 ± 0.94 ^a^	0.8620 ± 0.06 ^a^
24	49.72 ± 5.71 ^b^	3.12 ± 0.03 ^bc^	62.91 ± 0.47 ^b^	0.8970 ± 0.04 ^c^
48	33.72 ± 5.99 ^a^	2.98 ± 0.05 ^ab^	68.23 ± 1.13 ^c^	0.9807 ± 0.01 ^d^
72	29.36 ± 3.22 ^a^	2.81 ± 0.17 ^a^	72.92 ± 1.43 ^d^	0.8800 ± 0.09 ^b^

Different superscripts in the same column are significantly different (*p* < 0.05). TPC = total phenolic content, TFC = total flavonoids content.

**Table 4:** Influence of spontaneous fermentation on the colour attributes of finger millet biscuits.

Fermentation Period (h)	L*	a*	b*	Chroma	Hue Angle	ΔE
LBFMB						
0	28.50 ± 0.81 ^a^	7.89 ± 0.16 ^a^	7.41 ± 0.26 ^a^	10.82 ± 0.25 ^a^	43.20 ± 0.88 ^a^	0.00 ± 0.00 ^a^
24	30.72 ± 0.64 ^b^	7.82 ± 0.19 ^a^	8.18 ± 0.52 ^ab^	11.32 ± 0.50 ^ab^	46.25 ± 1.17 ^b^	2.72 ± 0.68 ^b^
48	31.15 ± 0.42 ^c^	8.09 ± 0.27 ^a^	8.77 ± 0.49 ^b^	11.93 ± 0.54 ^b^	47.30 ± 0.63 ^b^	2.97 ± 0.58 ^b^
72	35.58 ± 0.85 ^d^	8.08 ± 0.25 ^a^	11.04 ± 0.77 ^c^	13.68 ± 0.78 ^c^	53.76 ± 1.04 ^c^	7.96 ± 1.11 ^c^
DBFMB						
0	28.87 ± 0.57 ^a^	5.41 ± 0.18 ^a^	6.05 ± 0.36 ^a^	8.11 ± 0.34 ^a^	48.15 ± 1.46 ^a^	0.00 ± 0.00 ^a^
24	28.99 ± 0.81 ^a^	5.38 ± 0.19 ^a^	6.18 ± 0.47 ^a^	8.20 ± 0.48 ^a^	48.88 ± 1.16 ^a^	0.67 ± 0.31 ^a^
48	28.54 ± 0.54 ^a^	4.94 ± 0.34 ^a^	5.84 ± 0.81 ^a^	7.65 ± 0.82 ^a^	49.58 ± 2.33 ^b^	0.92 ± 0.35 ^b^
72	28.70 ± 0.40 ^a^	5.05 ± 0.69 ^a^	5.91 ± 1.24 ^a^	7.78 ± 1.39 ^a^	49.26 ± 2.00 ^b^	1.26 ± 0.16 ^b^

Different letters in the same column are significantly different (*p* < 0.05). L*, a*, b* and ΔE denotes lightness, redness, yellowness and total colour difference, respectively. LBFMB = light brown finger millet biscuits; DBFMB = dark brown finger millet biscuits.

**Table 5:** Influence of spontaneous fermentation on physical properties of finger millet biscuits.

Fermentation Period (h)	Diameter (cm)	Thickness (cm)	Weight (g)	Spread Ratio	Hardness (g)
LBFMB					
0	4.76 ± 0.05 ^b^	0.66 ± 0.05 ^a^	12.77 ± 0.92 ^c^	7.25 ± 0.53 ^c^	689.61 ± 2.70 ^a^
24	4.72 ± 0.08 ^ab^	0.66 ± 0.09 ^a^	12.69 ± 0.41 ^c^	7.25 ± 0.88 ^c^	1092.07 ± 3.55 ^b^
48	4.62 ± 0.08 ^a^	0.70 ± 0.07 ^ab^	12.53 ± 0.50 ^b^	6.66 ± 0.75 ^b^	1578.96 ± 1.93 ^c^
72	4.68 ± 0.13 ^ab^	0.76 ± 0.05 ^ab^	12.10 ± 1.57 ^a^	6.19 ± 0.61 ^a^	2212.79 ± 3.13 ^d^
DBFMB					
0	4.70 ± 0.10 ^b^	0.68 ± 0.08 ^a^	12.74 ± 1.09 ^c^	6.98 ± 0.76 ^d^	710.95 ± 2.11 ^a^
24	4.64 ± 0.13 ^b^	0.72 ± 0.04 ^a^	12.45 ± 0.75 ^b^	6.46 ± 0.29 ^c^	993.20 ± 2.41 ^b^
48	4.68 ± 0.13 ^b^	0.76 ± 0.05 ^a^	11.99 ± 0.75 ^a^	6.36 ± 0.62 ^b^	1474.97 ± 3.14 ^c^
72	4.54 ± 0.22 ^a^	0.74 ± 0.05 ^a^	11.99 ± 0.84 ^a^	6.05 ± 0.76 ^a^	2372.23 ± 1.25 ^d^

Different superscripts in the same column are significantly different (*p* < 0.05). LBFMB = light brown. finger millet biscuits; DBFMB = dark brown finger millet biscuits.

**Table 6:** Sensory evaluation of spontaneously fermented finger millet biscuits.

	Fermentation Period (h)			
LBFMB	0	24	48	72
Appearance	7.70 ± 1.56 ^a^	7.67 ± 2.44 ^a^	7.20 ± 2.52 ^a^	7.20 ± 1.37 ^a^
Colour	8.50 ± 2.20 ^a^	8.32 ± 1.15 ^a^	6.28 ± 1.30 ^b^	5.10 ± 1.45 ^c^
Aroma	7.20 ± 2.43 ^a^	7.15 ± 1.22 ^a^	6.25 ± 1.56 ^b^	6.10 ± 1.44 ^b^
Taste	7.56 ± 1.10 ^a^	7.10 ± 2.46 ^a^	6.70 ± 1.11 ^b^	6.50 ± 2.43 ^b^
Texture	6.40 ± 1.28 ^a^	6.18 ± 1.90 ^a^	6.10 ± 1.15 ^a^	5.44 ± 1.17 ^b^
Overall acceptability	7.80 ± 1.58 ^a^	7.34 ± 2.00 ^a^	7.00 ± 1.00 ^ab^	6.80 ± 1.58 ^b^
DBFMB				
Appearance	6.65 ± 1.44 ^a^	6.50 ± 1.53 ^a^	6.15 ± 1.65 ^a^	6.00 ± 1.76 ^a^
Colour	5.80 ± 1.34 ^a^	5.50 ± 1.33 ^a^	4.45 ± 1.52 ^b^	4.44 ± 1.74 ^b^
Aroma	6.70 ± 1.00 ^a^	6.45 ± 2.40 ^a^	5.98 ± 1.34 ^b^	5.95 ± 1.45 ^b^
Taste	7.20 ± 1.80 ^a^	5.90 ± 2.00 ^b^	5.40 ± 2.11 ^b^	4.10 ± 2.05 ^c^
Texture	6.40 ± 1.82 ^a^	5.80 ± 1.10 ^b^	5.56 ± 1.20 ^b^	4.40 ± 1.53 ^c^
Overall acceptability	6.67 ± 1.00 ^a^	5.38 ± 1.58 ^b^	5.35 ± 1.54 ^b^	4.90 ± 1.80 ^c^

Different letters in the same row are significantly different (*p* < 0.05). LBFMF = light brown finger millet biscuits; DBFMF = dark brown finger millet biscuits.

## Data Availability

The data presented in this study are available upon request from the authors.

## Supplementary Materials

The following supporting information can be downloaded at: https://www.mdpi.com/article/10.3390/foods11091265/s1, Figure S1: Scanning electron microscopy macrographs of light and dark brown finger millet biscuits; (a) Native biscuit; (b) Native dark brown FM biscuit; (c) 24 h fermented light brown fermented FM biscuit; (d) 24 h fermented dark brown FM biscuit; (e) 48 h fermented light brown FM biscuit (f) 48 h fermented dark brown FM biscuit; (f) h fermented dark brown FM biscuit; (g) 72 h fermented dark brown FM biscuit; (h) 72 fermented dark brown FM biscuit; PB= protein bodies; SG= starch granules; PLM = protein lipid matrix. FM = finger millet.
