# Supplementary material for: Nutritional Quality, Antioxidant, Microstructural and Sensory Properties of Spontaneously Fermented Gluten-Free Finger Millet Biscuits
Source: Foods. 2022 Apr 27;11(9):1265. doi: 10.3390/foods11091265 (PMC9105919; doi:10.3390/foods11091265)
Supplement: Supplementary file 1 [file foods-11-01265-s001.zip › foods-1632327-supplementary.pdf]

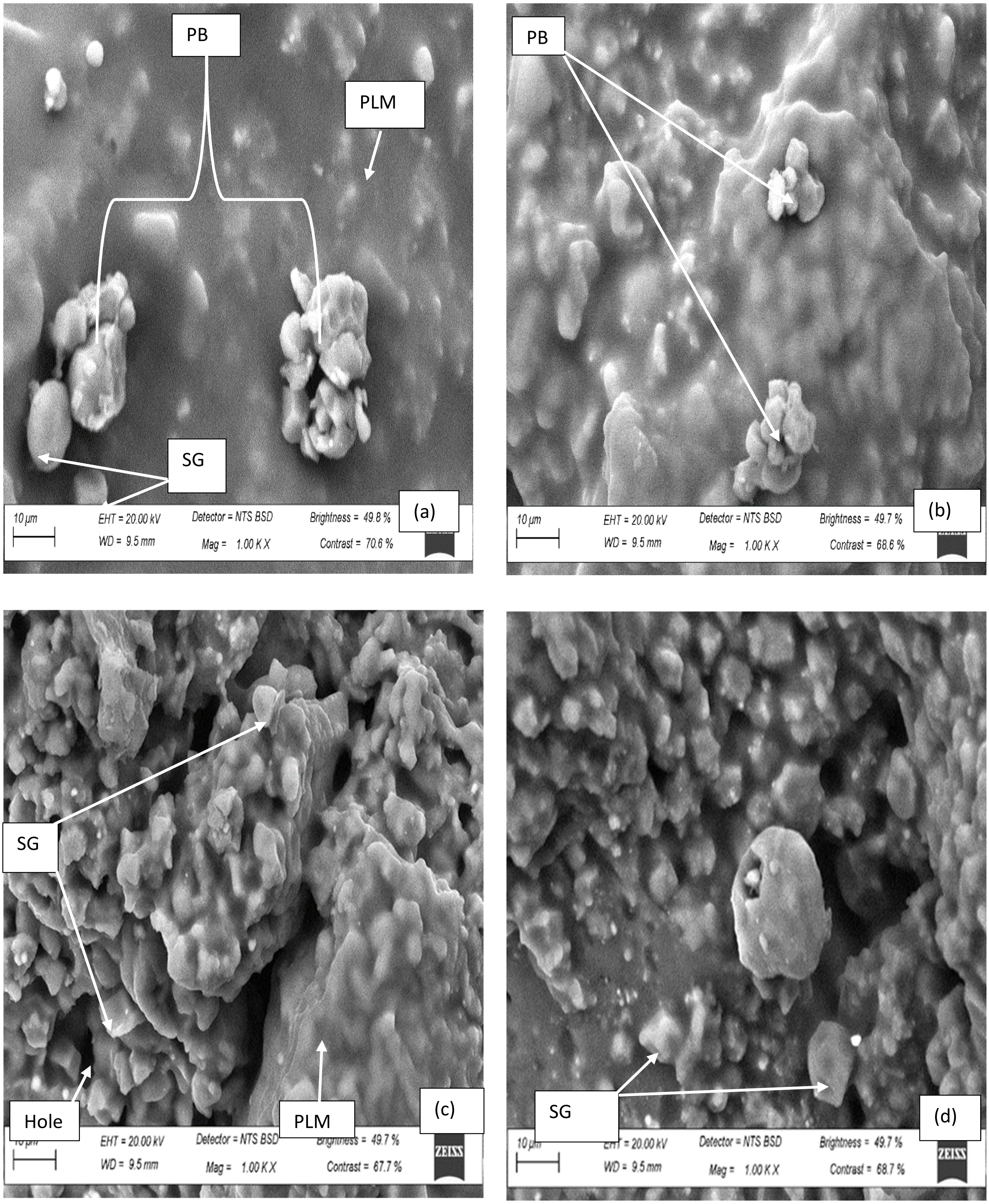

PB
PLM
SG
10 µm
EHT = 20.00 kV
WD = 9.5 mm
Detector = NTS BSD
Mag = 1.00 K X
Brightness = 49.8 %
Contrast = 70.6 %
(a)
PB
10 µm
EHT = 20.00 kV
WD = 9.5 mm
Detector = NTS BSD
Mag = 1.00 K X
Brightness = 49.7 %
Contrast = 68.6 %
(b)
SG
Hole
PLM
EHT = 20.00 kV
WD = 9.5 mm
Detector = NTS BSD
Mag = 1.00 K X
Brightness = 49.7 %
Contrast = 67.7 %
ZEISS
(c)
SG
EHT = 20.00 kV
WD = 9.5 mm
Detector = NTS BSD
Mag = 1.00 K X
Brightness = 49.7 %
Contrast = 68.7 %
ZEISS
(d)

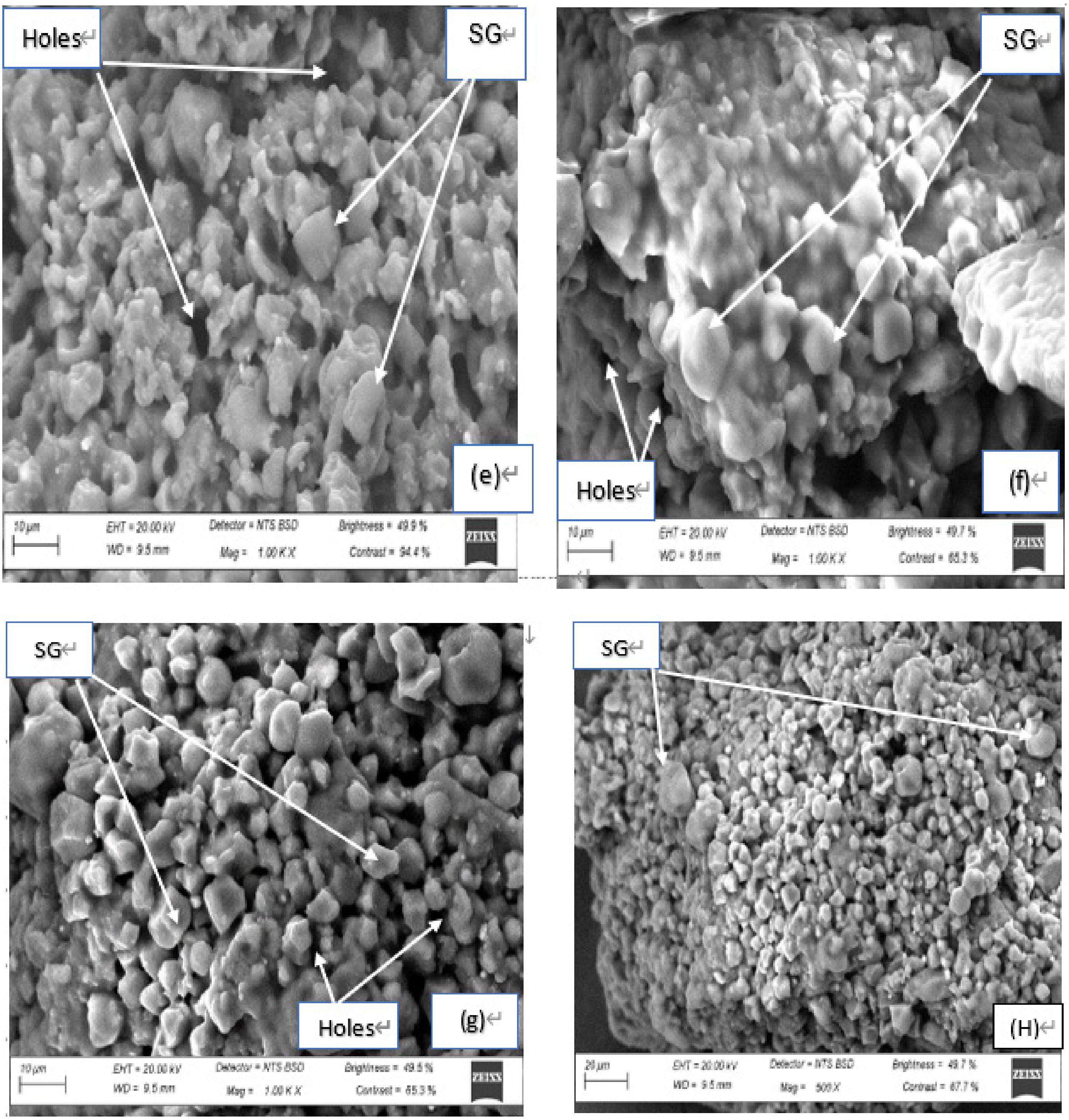

Figure S1: Scanning electron microscopy macrographs of light and dark brown finger millet biscuits; (a) Native biscuit; (b) Native dark brown FM biscuit; (c) 24 h fermented light brown fermented FM biscuit; (d) 24 h fermented dark brown FM biscuit; (e) 48 h fermented light brown FM biscuit (f) 48 h fermented dark brown FM biscuit; (f) h fermented dark brown FM biscuit; (g) 72 h fermented dark brown FM biscuit; (h) 72 fermented dark brown FM biscuit; PB= protein bodies; SG= starch granules; PLM = protein lipid matrix. FM = finger millet.
